# Reporting of somatic variants in clinical cancer care: recommendations of the Swiss Society of Molecular Pathology

**DOI:** 10.1007/s00428-024-03951-0

**Published:** 2024-10-23

**Authors:** Yann Christinat, Baptiste Hamelin, Ilaria Alborelli, Paolo Angelino, Valérie Barbié, Bettina Bisig, Heather Dawson, Milo Frattini, Tobias Grob, Wolfram Jochum, Ronny Nienhold, Thomas McKee, Matthias Matter, Edoardo Missiaglia, Francesca Molinari, Sacha Rothschild, Anna Bettina Sobottka-Brillout, Erik Vassella, Martin Zoche, Kirsten D. Mertz

**Affiliations:** 1 Clinical Pathology Division, Geneva University Hospitals, Geneva, Switzerland; 2 Institute of Medical Genetics and Pathology, Universitätsspital Basel, Basel, Switzerland; 3 TDS-Facility, AGORA Cancer Research Center, Swiss Institute of Bioinformatics, Lausanne, Switzerland; 4 Clinical Bioinformatics, Swiss Institute of Bioinformatics, Geneva, Switzerland; 5 Institute of Pathology, Lausanne University Hospital and Lausanne University, Lausanne, Switzerland; 6 Clinical Genomics Lab, Institut für Gewebemedezin und Pathologie, Inselspital, Bern, Switzerland; 7 Istituto Cantonale di Patologia EOC, Locarno, Switzerland; 8 Institut für Pathologie, Kantonsspital St-Gallen, St-Gallen, Switzerland; 9 Institut für Pathologie und Molekularpathologie, Universitätsspital Zürich, Zurich, Switzerland; 10 Oncology and Hematology, Cantonal Hospital Baden, Baden, Switzerland

**Keywords:** NGS reporting, Guidelines, Swiss Society of Molecular Pathology, Cancer

## Abstract

Somatic variant testing through next-generation sequencing (NGS) is well integrated into Swiss molecular pathology laboratories and has become a standard diagnostic method for numerous indications in cancer patient care. Currently, there is a wide variation in reporting practices within our country, and as patients move between different hospitals, it is increasingly necessary to standardize NGS reports to ease their reinterpretation. Additionally, as many different stakeholders—oncologists, hematologists, geneticists, pathologists, and patients—have access to the NGS report, it needs to contain comprehensive and detailed information in order to answer the questions of experts and avoid misinterpretation by non-experts. In 2017, the Swiss Institute of Bioinformatics conducted a survey to assess the differences in NGS reporting practices across ten pathology institutes in Switzerland. The survey examined 68 reporting items and identified 48 discrepancies. Based on these findings, the Swiss Society of Molecular Pathology initiated a Delphi method to reach a consensus on a set of recommendations for NGS reporting. Reports should include clinical information about the patient and the diagnosis, technical details about the sample and the test performed, and a list of all clinically relevant variants and variants of uncertain significance. In the absence of a consensus on an actionability scheme, the five-class pathogenicity scheme proposed by the ACMG/AMP guideline must be included in the reports. The Swiss Society of Molecular Pathology recognizes the importance of including clinical actionability in the report and calls on the European community of molecular pathologists and oncologists to reach a consensus on this issue.

**Supplementary Information:**

The online version contains supplementary material available at 10.1007/s00428-024-03951-0.

## Introduction

Over the past decade, the integration of high-throughput sequencing technologies (HTS) or next-generation sequencing (NGS) into precision oncology has created a diverse user landscape ranging from large academic centers to cantonal hospitals and private laboratories. This rapid development, driven by ever-decreasing sequencing costs and facilitated analysis schemes, has significantly expanded the capabilities of HTS/NGS, making sequencing with medium to large panels for the analysis of solid tumors, hematologic malignancies and cell-free DNA (cfDNA) commonplace [1]. However, this diversity implies differences in sequencing methods, bioinformatics pipelines and, most importantly, reporting methods. As HTS/NGS becomes a standard tool for identifying genomic alterations in cancer, the challenge has shifted to producing reports that are both comprehensive and concise. Gene panels currently cover up to hundreds of genes and may expand to the entire exome or genome in the forthcoming years, as seen in some European countries [2]. Furthermore, the targets of the analysis have evolved from point mutations to include copy number evaluation and the detection of gene fusions. These developments have resulted in increasingly complex reports, with tens to hundreds of variants to interpret and include in the report. In addition, it has become evident that reports should not only list relevant alterations, but also provide comprehensive information about the analytical methodology (e.g., the sequencing technology, gene panel or quality metrics used) and negative findings in certain types of cancer [3, 4].

The molecular pathology report, an important document containing sequencing results and functional variant annotations, is crafted by molecular pathologists, molecular biologists, and bioinformaticians in a collaborative effort. In Switzerland, public and commercial laboratories generate this report, which is then usually passed to pathologists, hematologists, and oncologists, who assess the clinical relevance of these alterations for patient care and translate them into clinical applications to provide information regarding the diagnosis, prognosis or drug sensitivity of the lesion. While some of these alterations are part of the standard of care, others require more complex interpretation, and the number of those alterations increases with the size of the sequencing panel employed. In an ideal scenario, a multidisciplinary molecular tumor board deliberates on the findings, integrating expertise from oncologists, pathologists, molecular biologists, bioinformaticians, and geneticists [5, 6]. This synergistic approach is essential due to the complexity of precision oncology, which leverages more precise diagnoses and an increasing number of targeted therapies to treat cancer [7]. The accurate interpretation of these reports demands familiarity with the latest advancements in cancer treatment and the relevance of the detected alterations, sometimes including potential cancer-predisposing germline mutations and variants of uncertain significance (VUS). Ultimately, the report may also be read by general practitioners and patients, underscoring the necessity for it to be exhaustive yet clear and concise [8].

Despite the existence of numerous guidelines for variant interpretation and reporting [9–15], there are inconsistencies between the reports from different Swiss institutions. In Switzerland’s healthcare system, where patients frequently receive care from various specialized institutions and seek second opinions, the need for standardized NGS reporting is paramount. Standardized reports will ensure that the information provided is easy to interpret, regardless of the healthcare provider. This will facilitate seamless care transitions and thorough understanding by all members of a patient’s care team. Such reports will accelerate collaborations by saving time and resources [16, 17], while avoiding potential oversights or errors. Over the past five years, significant discrepancies in the NGS reports produced by Swiss pathology laboratories have been identified through a series of surveys and discussions. By analyzing these discrepancies and the feedback from these surveys, we have developed and agreed on guidelines that reflect the collective expertise of Swiss molecular pathology professionals, taking into account the current legal framework and recommendations and tools available in the field. This initiative aims to create a basic structure for NGS reporting that promotes interoperability between healthcare organizations and professionals who rely on NGS data, in the context of solid tumors, hematologic malignancies, and cfDNA for patient care in Switzerland.

## Method

A round robin trial was conducted in 2017 by the Swiss Institute of Bioinformatics to assess the differences in variant calling practices and results across Switzerland. Six artificial NGS results (2 acute myeloid leukemias, 1 lung adenocarcinoma, and 3 rectal carcinomas) generated from IonTorrent data and targeted panels were sent to eight pathology and hematopathology institutes from six centers (Centre Hospitalier Universitaire Vaudois, Hôpitaux Universitaires de Genève, Istituto Cantonale di Patologia, Universitätsspital Basel, Inselspital Bern, Universitätsspital Zürich) and two academic facilities (SIB and Nexus at ETHZ) in Switzerland. Laboratories received VCF files containing the raw variants and had to annotate them, filter them and provide a somatic variant analysis report as done in their clinical routine. Out of 68 report elements examined, discrepancies in reporting practices were found in 48 (70.6%) of them (Supplementary Table S1). A Delphi process [18] was then initiated to reach a consensus on which items should be mandatory in a NGS report.

In 2020, an anonymous survey was sent to all members of the Swiss Society of Molecular Pathology and open to anyone involved in clinical NGS analyses in Switzerland (Supplementary Table S2). Each problematic reporting element was reviewed with the questions whether or not the element should be mandatory in a report, and whether or not the element should be described with a controlled vocabulary. Sixteen responses were collected from 13 pathology institutes, including three commercial laboratories:

*Centre Hospitalier Universitaire Vaudois (2 responses), Hôpitaux Universitaires de Genève (2 responses), Institute of Oncology Research, Istituto Cantonale di Patologia, Kantonspital Baselland, Kempf und Pfaltz AG, Luzerner Kantonspital, Pathologie Länggasse, Promed Laboratoire Médical SA, Swiss Institute of Bioinformatics (2 responses), Universitätsspital Basel, Universitätsspital Zürich, Viollier AG*.

A two-thirds majority was reached in 33 out of the 48 discrepancies observed during the round robin of 2017.

An expert group consisting of 1 to 2 members from the 5 university hospitals (Geneva, Lausanne, Bern, Basel, and Zurich) and the Istituto Cantonale di Patalogia (Locarno) was constituted. A first meeting where all 48 points were reviewed was held and resulted in only 11 items where no consensus had been reached. These were resolved by a second survey sent to the expert group (Supplementary Table S3) and a second meeting. It is noteworthy that the expert group decided not to address the issue of controlled vocabularies in the recommendation as it was difficult to reach consensus on most points and the issue was not considered to be of high importance.

## Recommendations

Note to the reader: Within this section, “must” denotes a required feature while “should” indicates an optional but recommended item. The use of “may” refers to an optional element that is neither recommended nor undesirable.

### General information and structure of the report

In agreement with the proposition from Schmid et al*.*[6], a report must contain the patient’s full name, date of birth, and biological sex. In addition, the name of ordering physician and the name and address of the laboratory that generated the report must also be present.

In terms of its structure, the report must have a dedicated section for the reported variants (a tabular format is recommended), a methodological section, and a summary of the findings either at the beginning or the end of the report. A short summary intended for patients and non-specialists may be included but is not required.

### Sample information

A report must include a unique specimen identifier, the diagnosis, and/or the clinical indication for the test. In addition, the report must enclose a description of the sample characteristics, including the collection date, the collection site (e.g., liver metastasis), the specimen type (e.g., FFPE or blood), and an estimation of the neoplastic cell content. Of note, the latter only applies to solid tumors and is not required in case of cfDNA analysis or hematologic malignancies. The adherence to international standards and ontologies for nomenclature is highly recommended (e.g., Oncotree [19], ICD-11 [20], and HL7 FHIR [21]).

### NGS assay information

The report must contain information on the NGS assay used, i.e., sequencing technology or platform, name of the panel, and version. Following the recommendation from AMP/ASCO/CAP [11], a list of all covered regions and the reference transcripts used to derive the HGVS consequences, and not just a list of genes, must be present in the report or available via a link to a webpage. A short description of the methodology and its limitations must be included. That comprises a list of all software leveraged to obtain the results (and their version number), the reference genome used and a list of all filters applied on the variants (e.g., “variants with an allelic frequency below 5% are discarded” or “variants present in more than 1% of the general population are not reported”). It is not mandatory to include a table with the unfiltered results. All information related to the methodology can be either written in a well-separated section within the report or as an appendix included to the report.

Besides generic information about the test, a quality statement about this particular analysis must be reported. We strongly recommend using quality scores and their associated thresholds; however, a simple statement is acceptable. While it is desirable to have metrics about coverage and depth on the regions of interest, it is not mandatory to list all regions insufficiently covered.

### Reported variants

In accordance with the AMP/ASCO/CAP recommendations [11], a report must contain all variants of clinical relevance and variants of uncertain significance (VUS). The only exception to this statement is in the case of hypermutated tumors (e.g., microsatellite instable or POLE-mutated) where it is acceptable to report the number of VUS without listing them all. However, the laboratory must ensure that the unreported VUS can be communicated upon request. For the sake of clarity, it might be desirable to sort variants so that the most pathogenic or actionable ones are read first. Negative results, i.e., absence of variants, should be reported for specific genes and pathologies. In particular, it is advised to report negative results for genes relevant to therapy in the given diagnosis. However, given the current lack of consensus on a list of genes and pathologies, the latter is not mandatory.

All reported variants must be described with the HGVS.c and HGVS.p notations along with their allelic frequency (VAF) in the sample. The three-letter code for amino acids is to be preferred, as per HGVS recommendations. A reference sequence, such as RefSeq or Ensembl transcript identifiers, must be provided with any HGVS.c notation and we recommend including it for the HGVS.p notation as well. It is not mandatory to indicate the exon number as different numbering systems could lead to misinterpretation, especially for VUS. The reporting of the genomic DNA sequence change (HGVS.g) is not recommended as the HGVS.c notation conveys the same information in a more concise manner. Quality metrics at the variant level are recommended, especially when the report only provides a general statement about the quality of the analysis without any quality scores.

### Reporting of potential germline variants

According to Switzerland’s law on genetic testing (Ordinance on Human Genetic Analysis; OHGA) [22], reporting of germline variants is only allowed in accredited laboratories authorized by the Federal Office of Public Health (FOPH). Germline variant analysis is usually performed in human genetics laboratories, while somatic variant analysis for oncology purposes is handled by molecular pathology laboratories. The practice of the latter still falls under the same overarching legal framework but is subject to less stringent regulations as its main purposes is to report somatic rather than germline variants that have different implications both for the patient and potentially his/her relatives. As most oncology NGS analyses do not use a paired non-tumoral sample to subtract germline variants, incidental findings may arise and must be reported as potential germline variants with a recommendation of genetic counseling if appropriate. If some variants are reported as potentially germline, then the report must contain a statement indicating that the germline status cannot be asserted with certainty, even if all evidence points towards a germline origin (e.g., a *TP53* variant with a VAF of 50% in a sample with very little tumor content). According to the Swiss law, the laboratory has the obligation to inform the prescriber, before the test is realized, that such findings may arise (OHGA, Art. 19) and patient consent has to be collected beforehand (OHGA, Art. 61 al. 4). Of note, the recent modification of the Swiss law on human genetics analyses states that no incidental findings can be reported if the test was ordered by a specialist other than a medical doctor such as a pharmacist (OHGA, Art 6 al. 3, Art. 7 al. 4, Art 8 al. 3). Therefore, in such cases, the variant should be removed from the report.

### Variant interpretation

Recent guidelines recommend the use of a classification system based on actionability rather than pathogenicity [9, 11, 23]. Currently, two major schemes are proposed: the AMP/ASCO/CAP tier system and the ESMO Scale for Clinical Actionability of molecular Targets (ESCAT) [11, 23]. While an actionability score is highly desirable, such assessment is difficult to implement in clinical routine. Until a consensus is reached on which system to use in Europe, the recommendation of the Swiss Society of Molecular Pathology is to continue to use the 5-class pathogenicity system described by the ACMG guidelines [24].

### Other reporting elements

Reports often contain complementary information, such as tumor mutation burden (TMB), microsatellite instability (MSI) and homologous recombination deficiency (HRD) scores, mutational signatures, clinical trials, references to scientific literature, public databases (e.g., COSMIC [25] or OncoKB [26]), or treatment recommendations. None of these are mandatory but any score reported must have its methodology described in details. For instance, the computation method used for TMB estimation should be present in the report together with the panel size. The reporting of gene fusions or copy number alterations that are part of an NGS analysis should be done according to the AMG/ASCO/CAP guideline with the updated nomenclature for gene fusions (e.g., *BCR*::*ABL1*) [11, 27].

## Discussion

The recommendations presented here reflect what a somatic NGS report edited by a Swiss laboratory must and should contain at present. The main topic of discussions during the elaboration of these guidelines was the implementation of an actionability score. While the importance and usefulness of providing such a score is recognized by all, we must highlight that the current guidelines are not satisfactory and do not fit the current clinical practice in Switzerland. The annotation of actionability is a moving target as results from a clinical trial or the approval of a novel drug may change drastically the actionability of a variant. The most striking example is the KRAS G12C variant that was not actionable until the approval of specific KRAS G12C inhibitors, which made it a potential pan-cancer biomarker [28]. The annotation process is time-consuming and requires knowledge of clinical trials and scientific literature, oncology guidelines, drug indications, and molecular pathology. The current organization of Swiss hospitals and pathology institute does not allow for the consolidation of this expertise in molecular pathology laboratories, which makes the implementation of actionability scoring difficult without a set of guidelines and resources fitting the Swissmedic framework (like publicly available databases).

The recent publication in 2022 by ClinGen/CGC/VICC of standard operating procedures for the classification of pathogenicity of somatic variants in cancer [9] is a welcome solution to an old problem but does not address the issue of actionability. To this end, the AMP/ASCO/CAP actionability tier system seems promising but cannot be implemented in Switzerland without adaptations. For instance, “FDA” needs to be replaced by its Swiss equivalent, “Swissmedic”, or possibly its European equivalent, “EMA”, as patients may be willing to travel to neighboring countries for treatment [15]. A recent study demonstrated that around 70% of US laboratories have implemented the AMP/ASCO/CAP guideline in 2022, while there is no evidence that the ESCAT is currently being widely used in Europe [29].

For Switzerland, it seems more rational to adopt a European system rather than an American one. However, commercial automated annotation systems that are available in Europe such as QIAGEN QCI or Sophia Genetics DDM platform tend to adopt the AMP/ASCO/CAP scheme instead of ESCAT. It may thus be difficult to have a harmonized actionability score within Switzerland or Europe. In Germany, the National Center for Tumor Disease (NCT) has developed its own scheme to address these issues [15].

To conclude, there is a need for the Swiss Society of Molecular Pathology to collaborate with the Swiss Group for Clinical Cancer Research (SAKK) to identify the most pertinent actionability scheme, to adapt it if needed, and to develop the tools needed to implement it in clinical routine. One of these tools might be a Swiss-wide database of actionable variants with well-defined processes to keep it up to date, in the spirit of the SoVad database [30], or an annotation software such as the AI-based CancerVar [31]. A collaboration might even be desirable at the European level. A consensus on the actionability of variants could also lead to a consensus, for a given pathology, on a minimal set of genes to be included in an NGS panel.

However, even in the absence of actionability scores in NGS reports, an optimal therapeutic choice can still be achieved by interpreting the NGS report in a molecular tumor board. Consisting of a panel of experts in precision oncology, genetics, molecular pathology and bioinformatics, these boards can provide a comprehensive opinion on a range of targeted therapies, possibly experimental, in light of the NGS results and the patient’s clinical history and condition. The Swiss Society of Molecular Pathology fully encourages and supports such discussions, especially for analyses based on large panels or those without any clear actionable targets. Due to the highly dynamic nature of the field, re-interpretation of an existing report by a molecular tumor board is also encouraged.

These guidelines describe an ideal toward which each laboratory should tend. However, some laboratories may have little control on the formatting of their reports or might be tied to their commercial provider. Such technical issues might hinder the implementation of these guidelines across the whole spectrum of NGS providers in Switzerland and incentives will be needed to achieve NGS reporting harmonization.

## Conclusion

Developing guidelines for NGS reporting practices through a Delphi process is a demanding endeavor. Our study represents the diversity of NGS analysis providers (both public and private) and the expert group did not succumb to the temptation to make recommendations that represent only the minimum of what each provider is already doing. The participation of a large number of Swiss molecular pathology laboratories in this initiative underlines the desire to create and establish a Swiss consensus in NGS reporting. With these guidelines, summarized in Table 1, we have set up the basic structure of an interoperable NGS report for all Swiss healthcare providers. To build on this effort, a follow-up study should be conducted to assess compliance with these recommendations, identify the blocking points, and further discuss the implementation of an actionability score based on the latest developments in the field.

**Table 1:** List of mandatory and optional items of an NGS report

**General information**	
* Mandatory*	*Optional*
• Patient full name, date of birth, and biological sex • Name of ordering physician • Name and address of laboratory • Diagnosis and/or clinical indication • Sample identifier • Sample collection site (e.g., liver) and collection date • Specimen type (e.g., FFPE) • Estimated neoplastic cell content	
**Methodology**	
* Mandatory*	*Optional*
• Name of panel and version with a list of gene names and covered regions • Sequencing platform/technology • Reference genome and transcripts • Description of methodology for variant detection, including any variant filters, and limitations • Description of methodology for tumor mutation burden (TMB), microsatellite instability (MSI) or homologous recombination deficiency (HRD) scores, if applicable • List of all software used	• The whole methodology section or part of it can be available via a web link
**Results**	
* Mandatory*	*Optional*
• General quality statement • A table with all relevant variants and all VUS	• Quality metrics with respect to depth and coverage • If POLE-mutated or MSI, the number of VUS can be reported instead of the complete table • Negative results on specific genes and pathologies • Treatment or clinical trials recommendations
**Reported variants**	
* Mandatory (for each variant in the report)*	*Optional*
• HGVS.c and HGVS.p notation with reference transcript • Allelic frequency in the sample • Pathogenicity (5-class) according to ACMG guidelines • A statement if the variant is suspected of being potentially germline (unless it is a situation where it is legally prohibited to report incidental findings)	• Reference sequence for HGVS.p • Quality metrics • Exon number • Actionability according to ESCAT or AMP/ASCO/CAP • References to public databases (e.g., ClinVar)

## Supplementary Information

Below is the link to the electronic supplementary material.

Supplementary file1 (XLSX 37 KB)
